# A Comprehensive Review of the Role of Artificial Intelligence in Obstetrics and Gynecology

**DOI:** 10.7759/cureus.34891

**Published:** 2023-02-12

**Authors:** Sagar N Malani, Deepti Shrivastava, Mayur S Raka

**Affiliations:** 1 Department of Obstetrics and Gynecology, Jawaharlal Nehru Medical College, Datta Meghe Institute of Higher Education & Research, Wardha, IND

**Keywords:** ultrasonography, postpartum period, artificial neural networks, gynecology, obstetrics, artificial intelligence in medicine

## Abstract

The exponential growth of artificial intelligence (AI) has fascinated its application in various fields and so in the field of healthcare. Technological advancements in theories and learning algorithms and the availability of processing through huge datasets have created a breakthrough in the medical field with computing systems. AI can potentially drive clinicians and practitioners with appropriate decisions in managing cases and reaching a diagnosis, so its application is extensively spread in the medical field. Thus, computerized algorithms have made predictions so simple and accurate. This is because AI can proffer information accurately even to many patients. Furthermore, the subsets of AI, namely, machine learning (ML) and deep learning (DL) methods, have aided in detecting complex patterns from huge datasets and using such patterns in making predictions. Despite numerous challenges, AI implementation in obstetrics and gynecology is found to have a spellbound development. Therefore, this review propounds exploring the implementation of AI in obstetrics and gynecology to improve the outcomes and clinical experience. In that context, the evolution and progress of AI, the role of AI in ultrasound diagnosis in distinct phases of pregnancy, clinical benefits, preterm birth postpartum period, and applications of AI in gynecology are elucidated in this review with future recommendations.

## Introduction and background

The digitalized computer system that mimics the processing of the human brain is termed artificial intelligence (AI). This intelligence system is systematized similarly in that the brain's neuron is arrayed with numerous neural nodes, which are known as neural networks [1]. The rapid markup of AI in the recent epoch has led to the proliferation of advancements in artificial neural networks (ANNs) that make interpreting multifactorial data simpler with the mathematical system [2]. These networks attain the most probable outcomes as the neurons are associated with numerous synapses that aid in transferring the data among the neurons back and forth. Assembling these manifold connections makes the computers imitate cognitive functions such as finding the appropriate solution to a problem, reasoning, etc. The multifarious advantages of AI have made it extensively used in the medical field for analyzing huge amounts of data. This aids in monitoring patients, prognosis, diagnosis, and disease prevention [3]. Over the past few decades, a massive rise in AI usage has been witnessed in healthcare systems. It has been evident from the statistical records that AI investments in healthcare systems are anticipated to rise 18 times by 2025. However, AI in healthcare systems requires collaboration and training among the partners for successful implementation. AI applications in healthcare include computer-aided fetal evaluators (CAFEs), cardiotocography (CTG), etc. System 8000 is a technology designed to monitor episodic changes in fetal movements and heart rate (FHR). On the other hand, neural networks have enabled the earlier detection of ovarian cancer. Also, trials have been made to foresee preterm labor by examining the electrohysterography (uterine electrical signals) [4]. Amidst all these, the implementation of AI in vitro fertilization (IVF) is a fecund technique in clinics in the recent era.

Comprehensively, AI supports practitioners and clinicians in decision-making with highly assured decisions. Nevertheless, it is momentous to know that AI is not an alternative for clinical purposes. By far and large, the reason AI has a rampant spread in clinical diagnosis and decision-making is the deaths due to erroneous prediction and diagnosis [5]. Thus, AI can help minimize such errors by enhancing predictive accuracy. Therefore, this review propounds the associated research, advantages, disadvantages, and potential applications of AI with prospects in obstetrics and gynecology.

## Review

Methodology

A literature review was undertaken with Google Scholar, Cureus, Comput Methods Programs Biomed, and the *International Journal of Gynecology & Obstetrics*. Terms considered for the key search included “Artificial Intelligence,” “obstetrics and gynecology,” “AI in obstetrics,” and “AI in gynecology.” All the suitable and relevant publications were found, with recent references (2012-2022) being included and publications (<2012) being excluded. Initially, the papers were chosen per the title relevance; this is followed by abstract screening and, when appropriate, the complete text. Moreover, the additional references cited by the paper were also evaluated and encompassed as necessary with overall refined papers as 20 that correspond well with the concept.

The need for the nexus of AI in obstetrics and gynecology

Obstetrics and gynecology are the debatable specialties that account for indemnity payments due to negligence claims. Besides litigation costs, socioeconomic consequences on a long-term basis due to medical errors have become detrimental [6]. Hypoxia-induced encephalopathy has become the most common confrontational event due to intrapartum fetal misinterpretation, which can be partially preventable. In addition, numerous poor outcomes and challenges have been recounted in gynecology, which is witnessed in gynecological oncology, where failed detection and prognosis of malignancy have been a major concern [7,8]. The conventional methodologies are considered inadequate in proffering treatment stratification on an individualized basis with various limitations. Infertility treatment has remained a major concern with conventional approaches. Thus, AI-assisted IVFs are instances of surging demands of AI in obstetrics and gynecology for enhanced success rates in treatment [9].

In addition, the rapid markup of advancements in genetic engineering in IVF practice raised the need for AI to enhance precision. Traditional methods have always been the most important tool in addressing healthcare issues among women through evidence synthesis, clinical trials, etc. Nevertheless, the gray areas present within the traditional approaches have been the reason for failure in providing appropriate solutions in clinical practices. Thus, AI has become one of the significant components of life today and so is its requirement in the field of medicine, especially in digital medicine. This lies in the fact that the progression of precision techniques has enabled accurate predictions in the healthcare domain as AI-based algorithms assist in meeting diagnostic challenges such as performance and efficiency in clinical services. These algorithms also improve clinical attentiveness in monitoring and treating complex diseases, controlling infections, etc. This shows that there is a clear need for the intervention of AI in obstetrics and gynecology. Hence, the upcoming sections of the review will encompass the use cases of AI in ultrasound diagnosis in the distinct phases of pregnancy, gynecological treatment, IVF, uterine myoma management, and uterine cancer.

Evolution and progress of AI in medicine

In the recent decade, there have been copious discussions about the AI position in the medicinal field, particularly focusing on big data management, assessment of algorithms, and medicolegal problems. The US Food and Drug Administration (FDA) has already endorsed various AI algorithms for the benefit of physicians and patients, and different organizations worldwide have followed the initiatives of the FDA [10].

**Figure 1:**
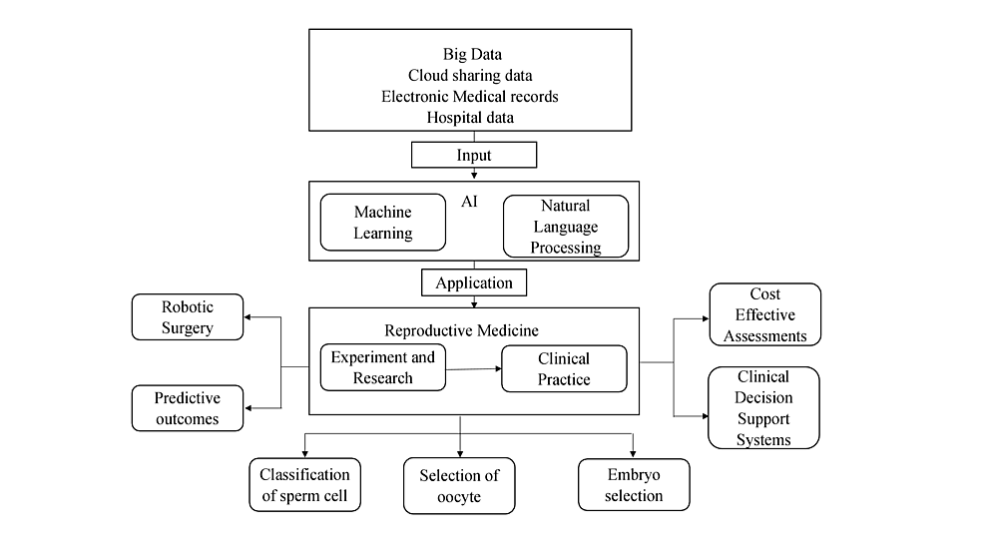
Role of AI in reproductive medicine. Source: [12]. AI, artificial intelligence

Researchers have written extensively about the advantages of AI applications that emphasize the technology's potential to enhance the accuracy of diagnoses, the entire clinical treatment process, and therapeutic efficacy. The applications of AI have assisted medical professionals and doctors in different domains, such as health information systems, syndromic and epidemic surveillance, geocoding of healthcare data, medical imaging, predictive model, and decision support system. The AI system is capable of providing health professionals with medical information with consistent and continuous real-time updates sourced from different textbooks, journals, clinical patients, and practices, which enables sophisticated and enhanced patient care and assists necessary inference for health outcomes prediction and health-risk assessment, specifically contributing to the field of gynecology and obstetrics [11]. The evolution of big data as an input to AI in the field of obstetrics and gynecology is shown in Figure 1.

Clinical benefits of obstetrics and gynecology through AI

The current usage of AI is addressed in obstetrics and gynecology to be the dominant tool in interpreting CTG and FHR, aiding in the determination of pregnancy complications and preterm labor, and reviewing the discrepancies in interpretation among clinicians. These benefits assist clinicians in decreasing the rate of infant and maternal mortality and morbidity. Furthermore, AI could be a prominent tool to generate algorithms to identify asymptomatic women with shorter cervical lengths (CLs) who were at preterm birth (PTB) risk. In addition, the major advantages of vast AI storage capacity could support the determination of preterm labor risk factors through extensive genomic data and multiomics, thereby reducing pregnancy complications, decreasing operative time, and aiding surgeons in training within a realistic setting [1]. Three-dimensional (3D) printers could offer materials that mimic real tissues and supports trainees to get practice with the realistic model. Further, 3-D imaging permits better deep perception than its two-dimensional counterparts, permitting the surgeon to generate preoperative plans by the dimensions and depth of tissues.

 Applications of AI in obstetrics

AI applications are outstanding because they resolve long-standing challenges in diagnosis and treatment. One such review concluded that this AI could augment the knowledge and support medical practitioners in their decision-making in gynecology and obstetrics [13]. The interpretation of CTG and fetal physiology could be facilitated through AI, limiting obstetrics' adverse impacts. However, this CTG interpretation may be prone to misrepresentation and human errors due to higher intraobserver and interobserver variability. The study aimed to delineate this deep learning (DL) and machine learning (ML) could be adjunct in fetal monitoring and objectively supports identifying the necessity of cesarean section during the care of intrapartum [14]. ML is a subfield of AI that broadly defines the machine's capability for imitating intelligent human behavior. 

Ultrasound Diagnosis in Distinct Phases of Pregnancy

AI-empowered ultrasonography possesses the potential to accelerate the utilization of medical ultrasound in different clinical settings with wider usage by medical personnel. Hence, the AI application in ultrasonography during pregnancy could support physicians in triaging and diagnosing the body of pregnant women. For instance, in obstetric pelvic and echocardiography ultrasonography, wherein visual analysis and measurement are essential, the video clips would offer the entire group of relevant structured data, permitting spatiotemporal analysis and increasing the benefits of ANNs [15].

Wu et al. explored the accuracy rate of ultrasonic diagnosis based on AI algorithms, during pregnancy period diagnosis, on those patients complicated with brain tumors [16]. The accuracy of diagnosis based on AI was 94.50%, and *K*-value was 0.99. In similar to this, another researcher Wu designed the Artificial bee colony algorithm, processed the Doppler ultrasound images that could greatly enhance the ultrasound images quality, decreases the noise of images, and highly improvise the capability of clinical diagnoses of the disease in different pregnancy phases, up to the birth of a child [17].

Significance of AI in the First Trimester of Pregnancy

During pregnancy, the placental images of patients having hypertension, if determined, deviate from those populations without hypertension. The inferences proved as a marker to predict hypertensive disorders of pregnancy (HDP), for it is a noninvasive, cost-efficient technique to promote future directions. Hence, the research explicates the utilization of AI to assess the variations in the placental ultrasound image texture of pregnant women with hypertension and normal outcomes. Therefore, this paves the approach to develop a textural feature extractor module, which could estimate adverse pregnancy outputs before going for clinical manifestation of the disease [18]. Likewise, another research example conducted a prospective and descriptive study for around 244 pregnant women in their first trimester of pregnancy. The enrolled women were asked particularly about using supplements of iron, folic acid, potassium iodide, and multivitamins during pregnancy. The ANN model having the pregnancy check variables and intake of iodized salt, iodized supplements, and iodine rich-foods might be utilized to predict the deficiency of iodine in the earlier pregnancy period, which aids experts in going for a feasible diagnosis [19].

Significance of AI in the Second and Third Trimesters

Diagnostic support tools relying on AI have exhibited higher performance in distinct medical dimensions. However, the clinical application of AI remained challenging due to a lack of the explanatory capability of AI decisions, referred commonly to as the black box problem. This problem makes it tedious to construct trust with those medical professionals. Nevertheless, visualizing the deep neural network (DNN) internal representation would maximize explanatory capability and improvise the medical professional's confidence level in AI decisions [20]. Sakai et al. applied the novel DL-based explainable graph chart diagram representation, which supports fetal cardiac ultrasound screening, which generally possesses a low rate of detection of congenital heart disease in their second-trimester stages because of difficulty in mastering the technique [20]. As a result, the screening performance using AI in the second and third trimesters for diagnosing pregnant women using the diagram representation improves from 96% to 97.50% for experts and then from 82% to 89% for fellow persons [21].

Further, the fetal ultrasound plane differs rapidly because fetal movement requires an algorithm capable of assessing moving images in a real-time environment. For the development and validation of the AI system, prenatal ultrasound diagnosis AI conduct system (PAICS) is proposed to determine various fetal intracranial abnormality patterns in standardized sonographic reference planes to screen for any malformations of the congenital central nervous system (CNS) [22]. Similarly, Burgos-Artizzu et al. assessed the performance of the AI method based on automated analysis of fetal-brain morphology of fetus upon standard cranial ultrasound section for predicting the gestational age parameters in II and III trimesters of pregnancy women fetuses [19]. The comparative assessment is explored by comparing with present formulas through standardized fetal biometry [20].

Preterm Birth

PTB is one of the leading causes of neonatal death. Predicting PTB in the first and second trimesters of pregnancy would assist in improvising pregnancy outcomes. Accordingly, research expounds on bringing out an efficient prediction model for PTB, depending on ANNs. Different studies aided that CL sonographic measurements would be utilized to predict PTB in the first pregnancy trimester period. Nevertheless, other researcher needs to delineate the CL capability to screen PTB. Compared with the logistic-regression algorithm, the benefits of DL and ML are that they have the potential to process high-dimensional patient data and the self-learning ability [23].

In line with this application of AI, selecting a viable embryo remained a primary challenge in other medical streams of IVF. This seems to be essential to estimate the outputs leading to a shorter pregnancy time, resulting in a live birth of a healthier child. Zaninovic and Rosenwaks​​​​​​​ claimed that the potentiality of AI would bring automatization, precision, and standardization to IVF that has provoked more enthusiasm and attained traction in commercial sectors. Moreover, the AI applications in embryology have attained significant attention and has exposed reliability to various areas of reproduction science. Additionally, AI-driven strategies have the possibility to be rapid, objective, and significant. Extensive utility of AI to assess the characteristics of patients like ovarian reserve, endocrine status, diagnosis test, endocrine status, and age will enhance the effectiveness of treatment and diagnosis of the disorders in reproductive system [24]. Through AI assistance and tools, these parameters promote the likely outcomes of successful IVF. The obstacles toward high sensitivity in the pregnancy period consist of different unknown parameters that result in successful IVF outcomes, which are necessary to teach AI. For this statement, larger datasets, including computer vision, were utilized to design the ANN model effectively to maximize the predictive capability. Few other previous attempts have been made with AI methods to assess the human oocytes, predict normal fertilization, and analyze the embryo's development to blastocyst (BL) stages. The methods even assess the implantation potential through static oocyte images in the pre- and postpregnancy periods [24].

Postpartum Period

Pelvic floor dysfunction (PFD) is another general gynecological disease. The major clinical manifestations are pelvic organ prolapse, sexual dysfunction, urinary loss, and fecal incontinence. From this perspective, a study explores the application benefit of ultrasound technology and rehabilitation training, depending on AI algorithm in postpartum pelvic organ prolapse recovery [25]. Hence, AI algorithms possess good impacts in the processing of ultrasonic images. The pelvic floor rehabilitation training had a better effect on postpartum nursing of pelvic organ prolapse patients. In addition, different consumer-grade, wearable devices, including smart rings and smartwatches, could track semicontinuous physiological measures such as body temperature, heart rate variability and normal heart rate count, and oxygen saturation and blood pressure. They also track other behavior measures such as quality of sleep, sleep duration, the relative location of patients, and their activity. The tracking process of those physiological parameters had obvious benefits for precise early pregnancy-related conditions determination, including gestational hypertension and preeclampsia. The most general digital technology and AI application in the pregnancy period are heart rate tracking, activity tracking, and diagnosis practices. However, integrating special tools would achieve more from preconception to postpartum. As an illustration, a better understanding of the metamorphosis of pregnancy (BUMP) research was propounded as a feasible longitudinal study focusing on attaining a deep understanding of pre-pregnancy symptoms and pregnancy symptoms of the patient's experiences through digital tools and ANN implementation [26].

Applications of AI in gynecology

In this modern era, the involvement of AI in gynecology is increased drastically; as diseases increase, there is a need to enhance the detection process. In that context, various researchers have utilized the benefits of AI in disease prediction. The most recent implementations of AI-based models in gynecology for the detection of endometrial carcinoma, IVF, uterine sarcoma, and cervical intraepithelial neoplasia and the development of an anticancer drug are given in Table 1.

**Table 1:** Application of AI in gynecology. AI, artificial intelligence; CAD, computer-aided design; LR, logistic regression; ANN, artificial neural network; CART, classification and regression tree; DL, deep learning; FIGO, International Federation of Gynecology and Obstetrics; LVSI, lymphovascular space invasion; DMI, Deep Myometrial Invasion; MRI, magnetic resonance imaging; CNN, convolutional neural network; PWI, perfusion-weighted magnetic resonance imaging; ROI, return on investment; DT, decision tree; CIN, cervical intraepithelial neoplasia; MS, mass spectrometry; FTIR, Fourier transform infrared spectroscopy; NMR, nuclear magnetic resonance; SSS-Net, sprint semantic segmentation network; SCB, sprint convolutional block

Reference	Year	Need		Methodology	Disease	Conclusion
[27]	2018	For enhancing the diagnostic accuracy of ultrasound analysis, there is a need for developing CAD mechanisms in ultrasound images.		Involved three different AI-based approaches such as LR, ANN, and CART utilized for comparing the diagnostic efficiency of endometrial carcinoma among postmenopausal women with endometrial width of 5 mm with irregular vaginal bleeding.	Endometrial carcinoma	AI, DL in particular, with enhanced specificity and sensitivity is a useful also powerful mathematical device that could be utilized in the health sector and significantly promote public health.
[28]	2017	FIGO, LVSI, and DMI are significant prognostic factors that could be employed for risk stratification. Most of these prognostic factors could only be examined in functioning specimens attained during wide-ranging staging. Thus, a comprehensive, noninvasive diagnostic approach is needed in clinical practice that could predict invasion and tumor stage.		A total of 137 women with the presence of endometrial cancer with a maximum diameter >1 cm were included and had undergone 1.5 MRI before the hysterectomy. Then, texture evaluation was executed with commercial research software, and the neighboring region of attention was manually delimited.	Endometrial carcinoma	The diagnosis of DMI, LVSI, and high-level tumors is efficiently accomplished with texture analysis and radiofrequency modeling-based MRI. The diagnostic outcome is equivalent to the most experienced radiologists.
[29]	2020	The texture feature-based MRI employed in CAD and MRI could be combined with AI, which differentiates clinical pathologic prognosticators before treatment.		The myometrial invasion of endometrial cancer in MRIs is detected by using DL with CNN.	Endometrial carcinoma	It is believed that AI has the potential for assisting radiologists to serve as a reasonable alternative to detecting the myometrial invasion depth of stage I endometrial carcinoma.
[30]	2019	To differentiate uterine sarcoma from leiomyomas, opinions on a semi-CAD framework based on PWI are needed.		Seven parameters are extracted from each ROI by the radiologists for characterizing the contrast agent dynamic. Consequently, the input has been fed into the DT ensemble classifier that classifies lesions into benign malignant uterine sarcoma or uterine leiomyoma.	Uterine sarcoma	It has been denoted that the proposed path is efficient for obtaining a promising discriminative power, which could be utilized with conventional MRI for differentiating sarcomas from myomas.
[31]	2019	The automatic segmentation and detection of an irregular region in the cervical image plays a vital role in the diagnosis of cervical cancer.		Diffuse reflectance is developed, and system analysis is accomplished based on Lab-Windows (National Instruments Corp., Austin, TX, USA) development software and the MariaDB (MySQL, Cupertino, CA, USA) database. Then, an ANN system based on a spectral database has been implemented for differentiating cervical tissue from normal. Then, nude mouse-tumor-model and human-volunteer tests have been involved.	CIN	The outcome found that the hemoglobin rate in unit tissue increased during cell proliferation, increasing the light absorption coefficient. It has been proved that the system could categorize CIN from normal cervical, and it can be implemented in screening preinvasive cervical lesions.
[32]	2019	AI plays a vital role in drug research development.		Synthesized amides and esters that were two Gallic acid derivatives set. New compounds are characterized by spectral data like MS, FTIR, and NMR. These compounds were tested in vitro by the A2780 cell line. A novel approach with the presence of the Hill function is employed for characterizing the optimal anticancer activities. Also utilized was SVM combined with the pharmacodynamics modeling paradigm to verify the efficiency of anticancer compounds.	Anticancer drug	The outcome showed that all the evaluated compounds were biocompatible and resulted in strong anticancer characteristics against ovarian cancer cells - A2780. It has been denoted that the method might efficiently infer the in vitro outcome of the leading compounds produced in both the preclinical and in vivo research.
[33]	2022	The blastocyst viability is evaluated by an embryologist through a manual microscopic process of its components such as zona pellucida, trophectoderm, inner cell mass, and blastocoel. Along with the DL's success in the medical diagnosis sector, semantic segmentation has a high potential for detecting crucial human blastocyst components in computerized evaluations.		An SSS-Net is employed for the detection of blastocyst components for the analysis of embryology. The SSS-Net utilized the SCB that involves asymmetric kernel convolutions in combination with depth-wise separable convolution.	IVF	The introduced approach could be employed for verifying blastocysts' morphological properties for an efficient IVF procedure.
[34]	2021	IVF prediction is an imperative achievement in assisting reproduction, considerably aiding infertile couples, communities, and health systems.	Developed integrated omics and AI to enhance the success rate of treatment. Initially, recorded the subfertile lifestyle of couples and demographic parameters along with IVF cycle characteristics; then evaluated and measured the transcriptomics, biomarkers, and metabolomics and conducted deep ML assessment of the sperm, oocyte, and embryo. Finally, the development of the ANN system for increasing objectivity and accuracy compared with a conventional approach to improve the success rate of IVF cycles followed by IVF failure.		IVF	It has been expected that the evolution of the reproductive, simple, and cheap system could be employed in the everyday practice in IVF units, particularly after the large-scale valuation of metabolomics and transcriptomics that would reduce the cost of tests and make them obtainable in routine use.

AI has been widely employed in gynecology-based studies, as given in Table 1. The research finding has enumerated that the implemented models outperform and their findings are mostly equivalent to the most experienced medical professionals.

Research gap

Currently, the most challenging difficulty that clinical staffs frequently face in gynecologic practice is the differential analysis of malignant and benign adnexal masses using ultrasound images. The transvaginal ultrasound was the most beneficial path for identifying differences and has been thought to be the first-line imaging system. Moreover, it has chiefly relied on the experienced examiner, i.e., the main flaw of this equipment is that its diagnostic enactment was principally based on the biased impression of investigative clinical sonographers [27]. Moreover, it has been pointed out that the AI was more probably to result from misinterpretations in patients with coexisting polypoid tumors (or) benign leiomyomas [29]. With the individual variations of cancer patients and the multidrug conflict development, numerous patients with gynecological cancer have poor drug sensitivity follow-on unacceptable clinical treatment [32].

Recommendations 

At present, significant achievements have been accomplished in the application of AI in gynecology and obstetrics, but the effectiveness and universality of various models still need further exploration. Various skills have been explored in this study to solve the predicament of restricted accuracy, such as developing ensemble algorithms [35] by utilizing time series data as a validation set [36] or ultrasound videos [37] or by focusing features in harmonizing imaging modalities [38]. Additionally, with the continuous modification and optimization of algorithms, the concepts and logic behind these approaches should be known, not only by the algorithm developers but also by the medical professionals. Thus, they could standardize or eliminate subjective bias to avoid misdiagnosis for achieving fair, unified, and objective generalization standards. In another context, AI-based obstetric ultrasound technologies have been progressively on stage with a significant role in social and education services. The fetal-ultrasound telemedicine facility could link up the expert in the fetal clinical center and the remote obstetric unit that could facilitate high-quality ultrasound analysis and professional consultations and reduce the traveling time cost-efficiently. However, the technology was shown to be helpful in transnational consultation. As obstetric ultrasound remained unobtainable in numerous rural areas and developing countries, diagnostic and telemedicine could upsurge the admittance of obstetric ultrasound diagnosis in low-resource settings. Thus, this area needs to be researched further. The detection model needs to be extended further for analysis with other clinical images and suggested to attempt network optimization in mobile platforms [33]. Regardless of being an auspicious moment for AI, some problems still need to be solved in the future. So far, AI is still immature, in its start-up stage, and is still not an individual procedure. Medical professionals are still suggested to operate AI adequately for engendering their optimization and hypothesis of AI application in clinical practice.

## Conclusions

AI is primarily well-defined as the study of algorithms. This repeated and massive exploration provides machines with the capability of performing and reasoning cognitive purposes. Thus, machines can recognize objects, make decisions, and solve problems. AI has been developed as the most significant area in various industries, resulting in incredible potential. The streamlined efficiency and predictive performance related to disease diagnosis using AI, chiefly in clinical imaging errands, are equivalence with or even exceed that of doctors, and they are capable of the benefits of being determined and ensuring stable characteristics. AI has attained equivalent performance with that of medical experts in a particular medical sector, and obstetrics and gynecology are among them. Thus, this study reviewed the role of AI in obstetrics and gynecology. It also comprises the conventional clinical process for obstetrics and gynecology and the progress and evolution of AI in medicine. The application of obstetrics and gynecology was also highlighted in the study. Thus, with technological development and interdisciplinary incorporation, AI could propose much more in obstetrics and gynecology. 
